# Sexual and gender minority identity in undergraduate medical education: Impact on experience and career trajectory

**DOI:** 10.1371/journal.pone.0260387

**Published:** 2021-11-19

**Authors:** Josef Madrigal, Sarah Rudasill, Zachary Tran, Jonathan Bergman, Peyman Benharash

**Affiliations:** 1 Cardiovascular Outcomes Research Laboratories (CORELAB), Division of Cardiac Surgery, David Geffen School of Medicine, University of California, Los Angeles, California, United States of America; 2 Department of Urology, David Geffen School of Medicine, University of California, Los Angeles, California, United States of America; University of Perugia: Universita degli Studi di Perugia, ITALY

## Abstract

**Introduction:**

The wellbeing of sexual and gender minority (SGM) medical students and the impact of their experiences on career trajectory remain poorly understood. The present study aimed to characterize the incidence of mistreatment in SGM trainees as well as general perspectives on the acceptance of SGM individuals across medical and surgical specialties.

**Methods:**

This was a cross sectional survey study of all actively enrolled medical students within the six University of California campuses conducted in March 2021. An online, survey tool captured incidence of bullying, discrimination, and suicidal ideation as well as perceived acceptance of SGM identities across specialties measured by slider scale. Differences between SGM and non-SGM respondents were assessed with two-tailed and chi-square tests. Qualitative responses were evaluated utilizing a multi-stage, cutting-and-sorting technique.

**Results:**

Of approximately 3,205 students eligible for participation, 383 submitted completed surveys, representing a response rate of 12.0%. Of these respondents, 26.9% (n = 103) identified as a sexual or gender minority. Overall, SGM trainees reported higher slider scale scores when asked about being bullied by other students (20.0 vs. 13.9, P = 0.012) and contemplating suicide (14.8 vs. 8.8, P = 0.005). Compared to all other specialties, general surgery and surgical subspecialties had the lowest mean slider scale score (52.8) in perceived acceptance of SGM identities (All P < 0.001). In qualitative responses, students frequently cited lack of diversity as contributing to this perception. Additionally, 67.0% of SGM students had concerns that disclosure of identity would affect their future career with 18.5% planning to not disclose during the residency application process.

**Conclusions:**

Overall, SGM respondents reported higher incidences of bullying and suicidal ideation as well as increased self-censorship stemming from concerns regarding career advancement, most prominently in surgery. To address such barriers, institutions must actively promote diversity in sexual preference and gender identity regardless of specialty.

## Introduction

Over the past several decades, social inequities and discrimination against individuals identifying as sexual or gender minorities (SGM) have garnered significant national attention. Although acceptance of SGM in the United States has generally increased [1], disparities in the healthcare setting persist. Historically, SGM patients and their families have encountered barriers to care, perpetuating health inequalities in this population [2–4]. Furthermore, SGM physicians have previously described their individual struggles with discrimination at different stages of training [5–8]. A survey of SGM physicians found that a majority have faced or witnessed unacceptable behaviors including derogatory comments, social isolation, harassment, and denied referrals from their non-SGM colleagues [9]. Such experiences in the workplace may amplify minority-stress related to identity concealment and fear of rejection, which adversely impact wellbeing and professional standing [10].

Discrimination relating to sexual orientation and gender identity likely permeates throughout undergraduate medical education. In a 1996 study, one in four residency program directors admitted bias against SGM applicants who openly expressed their identities [11]. Another group reported that 16.2% of surgeons and 12.1% of obstetricians/gynecologists were opposed to SGM trainees entering their respective specialties [12]. More recent data from the 2016 and 2017 Association of American Medical Colleges (AAMC) Graduation Questionnaires revealed that SGM medical students were more likely to experience mistreatment [13], and subsequently, report higher rates of burnout [14]. Adversities encountered at this stage of training are thought to significantly impact choice of specialty [15] as well as the decision to disclose one’s sexual orientation and gender identity during the residency application process [16].

Although multiple studies have assessed the implication of SGM identity on residency experience and career advancement of graduate medical trainees, few have explored this topic in the undergraduate phase of medical education. The present study characterizes SGM medical students’ experiences with bullying, discrimination, and identity disclosure as well as the general perceptions of undergraduate medical trainees in regards to SGM acceptance among various specialties. We hypothesized that SGM medical students would report increased incidence of mistreatment and that specialties with a history of discrimination against SGM trainees, such as surgery and obstetrics/gynecology, would be viewed as least accepting.

## Methods

This was a cross-sectional study of all actively-enrolled medical students within the University of California. Approximately 3,205 students across six campuses (Davis, Irvine, Los Angeles, Riverside, San Diego, San Francisco) were eligible for participation. An online, anonymous survey tool through a secure form was made available to eligible participants from March 2, 2021 to March 21, 2021 in collaboration with each institution’s Office of Student Affairs.

Survey design utilized a comprehensive, deliberate and multistage approach. Following a systematic review of previously published surveys assessing sexual and gender minority trainees [17, 18], a preliminary survey was drafted with the assistance of content experts experienced in qualitative research. The preliminary tool was subsequently distributed to medical students outside of the University of California system as well as medical and surgical residents for feedback regarding the content and language of the instrument. The finalized survey was published into the Research Electronic Data Capture (REDCap) database for distribution. Participants provided consent prior to initiating the survey. To ensure their anonymity, no unique identifiers were collected. Responses were only accessible to the authors and were not made available to the participating institutions’ Offices of Student Affairs. Given the de-identified nature of the survey, the study was deemed exempt from full review by the Institutional Review Board at the University of California, Los Angeles.

The survey comprised four sections (S1 Appendix). The first assessed student demographics including sexual orientation and gender identity as well as age, year of medical school training, race, relationship status, parental income, and intended residency specialty. Respondents were classified as SGM if they marked their sexual orientation or gender identity as “bisexual,” “gay/lesbian,” “queer,” “transgender,” “non-binary,” or “other.” The second section utilized slider scales [19, 20] to assess each participant’s perceptions and personal comfort level in pursuing any of eight specialties. Training fields included anesthesiology, emergency medicine, family medicine, internal medicine, neurology, obstetrics and gynecology, pediatrics, general surgery or surgical subspecialties. Respondents were instructed to quantify how accepting of SGM students attendings were as well as how comfortable they would be in applying to residency in each of the aforementioned specialties. Scales ranged from 0 to 100 (0 = “Never Accepting/Comfortable”, 50 = “Sometimes Accepting/Comfortable”, 100 = “Always Accepting/Comfortable”). The third section also utilized slider scales to assess the frequency at which participants experienced discrimination or bullying as well as contemplated leaving medical school or committing suicide during their training. Slider scale values are as follows: 0 = “Never”, 50 = “Sometimes”, 100 = “Always”. The final section was administered only to participants identifying as SGM and examined their experiences with identity disclosure during medical school and the residency application process. Students were able to elaborate on their responses using free-text sections throughout the survey instrument.

Categorical variables are reported as frequencies with proportions while continuous variables are reported as means with standard deviation. Mean slider scale scores were calculated to assess perceived acceptance and level of comfort as well as frequency of experiencing mistreatment. To assess differences between SGM and non-SGM participants, univariate comparisons were performed using the two-tailed and chi-square tests for continuous and categorical variables, respectively. A p-value less than 0.05 was considered significant. Stata 16.1 (Statacorp, College Station, TX) was used to perform all statistical analysis. Qualitative responses were assessed using a multi-stage, cutting-and-sorting technique as described previously [21, 22]. Authors JM, SR, and ZT reviewed these responses independently and sorted them into four general themes.

## Results

A total of 383 University of California medical students completed the survey, representing a response rate of 12.0%. Respondents had an average age of 26.4 years, ranging from 22 to 45 years (Table 1). Students at all levels of undergraduate training as well as individuals on leaves of absence were represented in the study population. Overall, participants had a wide range of intended specialties with general surgery or surgical subspecialties (18.3%), internal medicine (14.1%), and family medicine (9.7%) being most common. Of 383 respondents, 26.9% (n = 103) were classified as a sexual or gender minority. There were no significant differences in the distributions of age, year of medical school training, race, parental income, or intended specialty between SGM and non-SGM students.

**Table 1:** Participant demographics stratified by sexual orientation and gender identity.

	Overall (n = 383)	Non-SGM^a^ (n = 280)	SGM (n = 103)	*P-Value*
**Age, mean (SD)**	26.4 (2.8)	26.3 (2.8)	26.6 (2.7)	0.29
**Level of Education, n (%)**				0.54
MS1	101 (26.4)	78 (27.9)	23 (22.3)	
MS2	66 (17.2)	46 (16.4)	20 (19.4)	
MS3	75 (19.6)	54 (19.3)	21 (20.4)	
MS4	124 (32.4)	87 (31.1)	37 (35.9)	
Leave of Absence/Other	17 (4.4)	15 (5.4)	2 (1.9)	
**Race, n (%)**				0.89
White	136 (35.5)	102 (36.4)	34 (33.0)	
Black	19 (5.0)	15 (5.4)	4 (3.9)	
Latinx/Hispanic	39 (10.2)	27 (9.6)	12 (11.7)	
Asian or Pacific Islander	142 (37.1)	103 (36.8)	39 (37.9)	
Other or Mixed Race	47 (12.3)	33 (11.8)	14 (13.6)	
**Parental Income, n (%)**				0.96
$0–$50,000	111 (29.0)	80 (28.6)	31 (30.1)	
$50,000–$100,000	85 (22.2)	61 (21.8)	24 (23.3)	
$100,00–$250,000	115 (30.0)	86 (30.7)	29 (28.2)	
> $250,000	72 (18.8)	53 (18.9)	19 (18.5)	
**Relationship Status, n (%)**				0.029
Single	131 (34.2)	91 (32.5)	40 (38.8)	
In A Relationship	250 (65.3)	189 (67.5)	61 (59.2)	
Other	2 (0.5)	0 (0)	2 (1.9)	
**Specialty of Interest, n (%)**				0.13
Anesthesiology	16 (4.2)	13 (4.6)	3 (2.9)	
Emergency Medicine	35 (9.1)	29 (10.4)	6 (5.8)	
Family Medicine	37 (9.7)	28 (10.0)	9 (8.7)	
Internal Medicine	54 (14.1)	44 (15.7)	10 (9.7)	
Neurology	8 (2.1)	6 (2.1)	2 (1.9)	
Obstetrics and Gynecology	28 (7.3)	17 (6.1)	11 (10.7)	
Pediatrics	27 (7.1)	21 (7.5)	6 (5.8)	
Psychiatry	33 (8.6)	17 (6.1)	16 (15.5)	
Surgery or Surgical Subspecialties	70 (18.3)	52 (18.6)	18 (17.5)	
Undecided	46 (12.0)	32 (11.4)	14 (13.6)	
Other	29 (7.6)	21 (7.5)	8 (7.8)	

^a^SGM—Sexual or Gender Minority.

**Fig 1:**
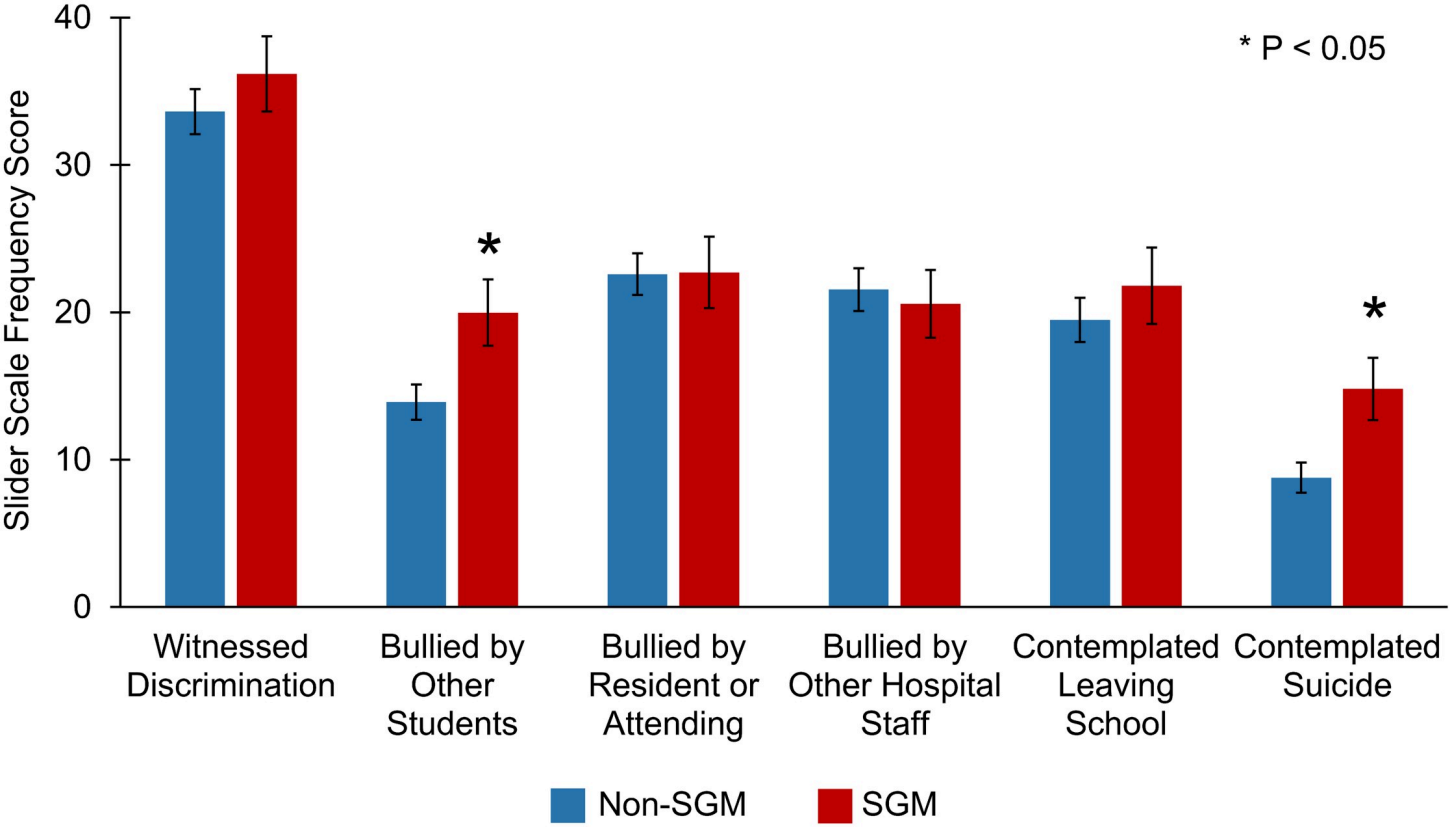
Work environment and mental health scales stratified by SGM identity. Values are as follows 0 = “Never”, 50 = “Sometimes”, 100 = “Always”. *P-Value < 0.05.

**Fig 2:**
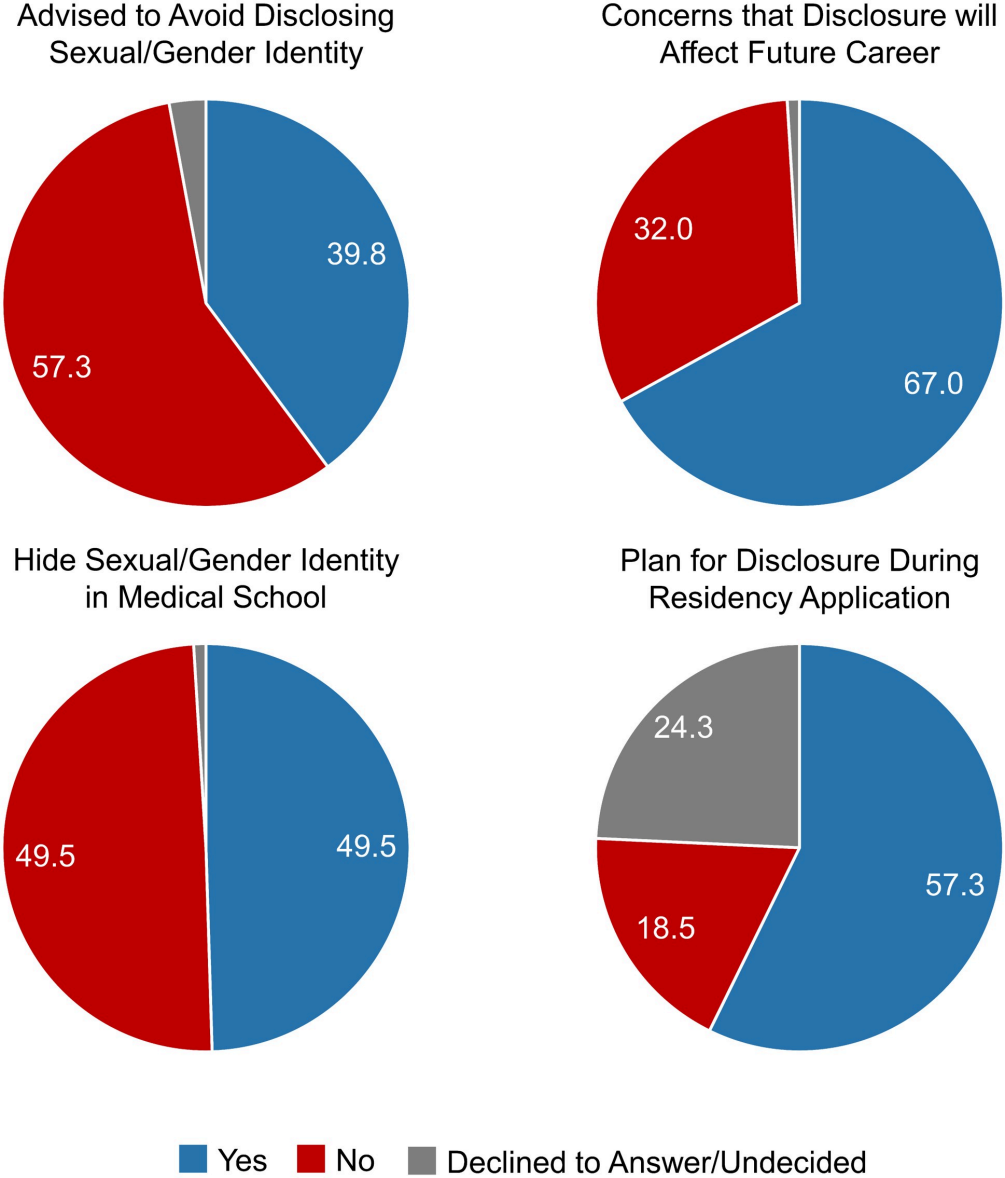
Barriers encountered by students classified as sexual and gender minorities. Participants were directed to these questions if they identified as a sexual or gender minority. Responses included Yes, No, Decline to Answer, and Undecided.

Overall, SGM trainees more frequently experienced bullying by other students (20.0 vs. 13.9, P = 0.012) compared to their non-SGM peers (Fig 1). Furthermore, these trainees more often contemplated suicide (14.8 vs. 8.8, P = 0.005). In assessing their experiences with self-censorship, 39.8% (n = 41) of SGM respondents had previously been advised to avoid disclosing their sexual or gender identity with 67.0% (n = 69) having concerns that disclosure would affect their future career (Fig 2). Additionally, 49.5% (n = 51) of students classified as SGM made efforts to hide their identities while in medical school with 18.5% (n = 19) planning to not disclose during the residency application process.

**Fig 3:**
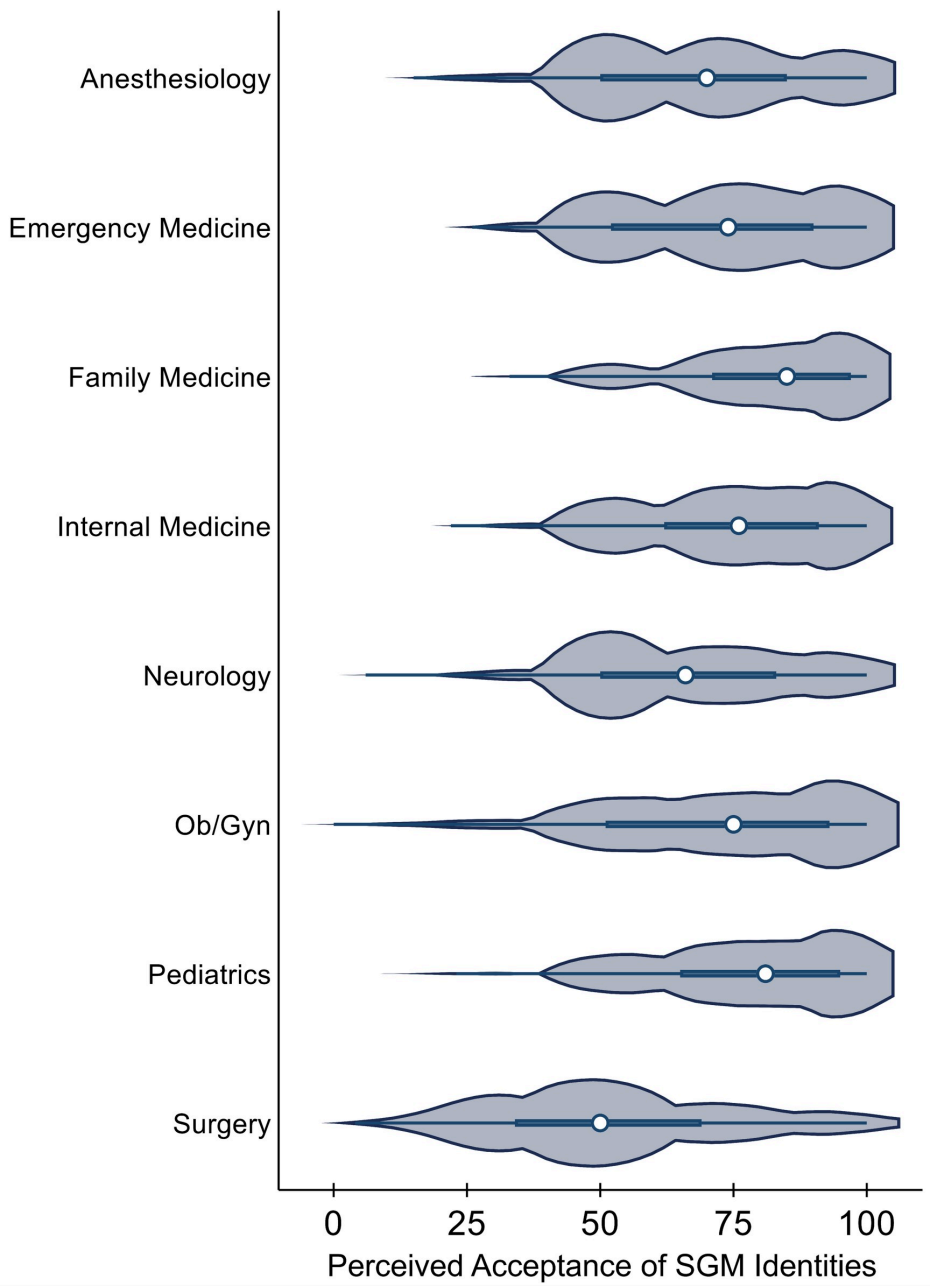
Perceived acceptance of sexual and gender minority identities amongst medical and surgical specialties. Values are as follows: 0 = “Never Accepting”, 50 = “Sometimes Accepting”, 100 = “Always Accepting”. Dots depict medians while bars depict interquartile ranges. Kernel Density is represented on this violin plot.

**Fig 4:**
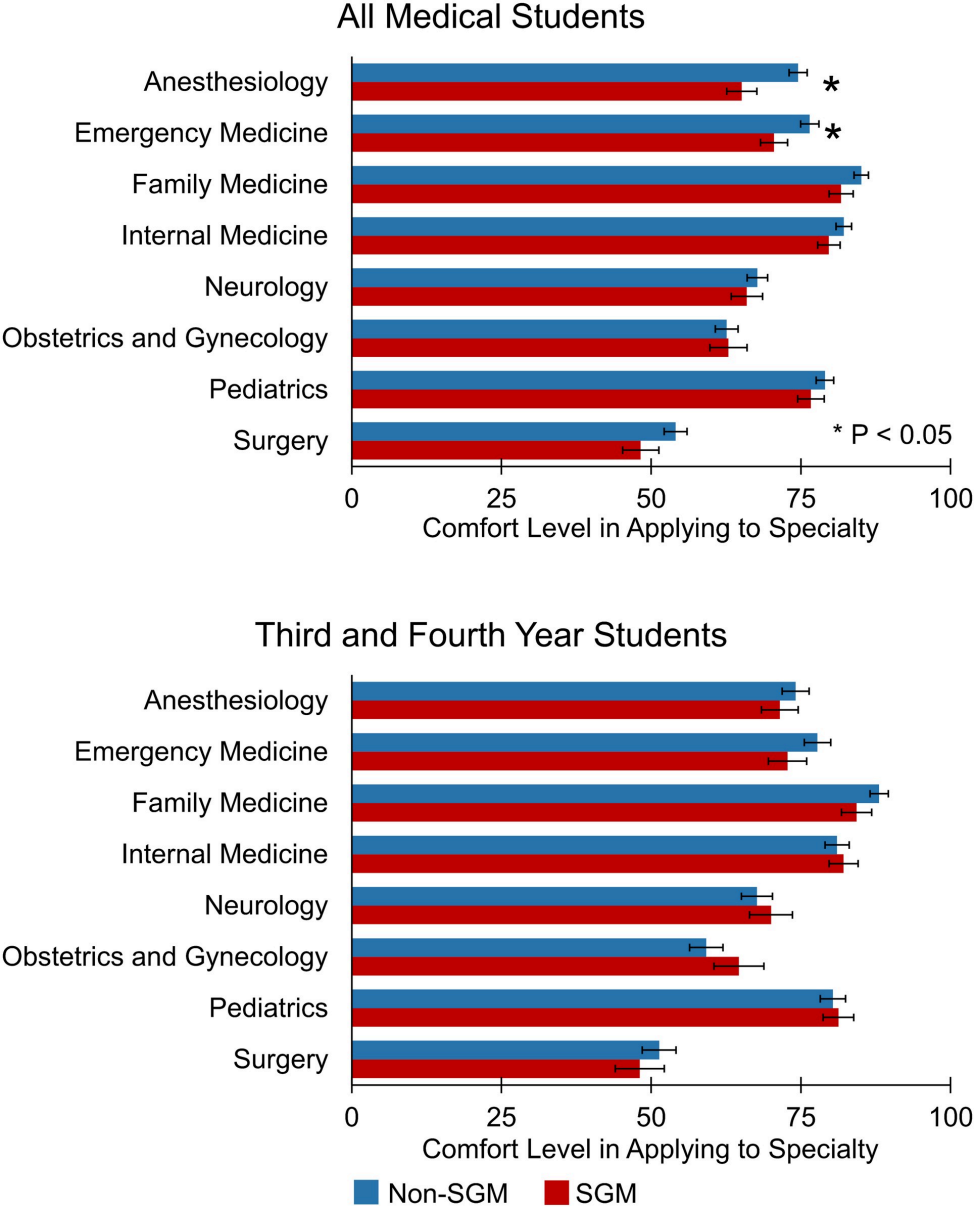
Level of comfort in applying to medical and surgical specialties stratified by SGM identity. Values are as follows: 0 = “Never Comfortable”, 50 = “Sometimes Comfortable”, 100 = “Always Comfortable”. *P-Value < 0.05.

Compared to all other specialties, general surgery and surgical subspecialties scored the lowest (52.8) in perceived acceptance of SGM identities (All P < 0.001) (Fig 3). This was followed by neurology (66.9) and anesthesiology (68.7). In general, respondents identifying as SGM were less comfortable than their non-SGM colleagues in applying for residency to anesthesiology (65.1 vs. 74.5, P = 0.001) and to emergency medicine (70.5 vs. 76.5, P = 0.038) (Fig 4A). On subpopulation analysis of third- and fourth-year medical students, however, no between-group differences were observed in level of comfort pursuing training in any of the specialties assessed (Fig 4B).

Four central themes were identified from qualitative responses. In general, students (1) viewed specialties that lack diversity as less accepting of SGM identities, (2) witnessed or personally experienced misgendering, (3) heard explicit comments regarding individuals of SGM identity from attendings, residents, and other hospital staff, and (4) viewed SGM identity as a barrier to career advancement. Theme 1 generated the most comments with students citing lack of sexual and gender as well as racial diversity:

“Many surgical specialties are male-dominated and tend to primarily be white males. The most homophobic and transphobic comments I have ever witnessed were during surgical rotations.”

Furthermore, respondents noted that this lack of diversity subsequently led to values incongruent to those of trainees:

“I do not see my own identity or beliefs reflected in attendings and residents in surgery. Surgical culture tends to be blunt and often politically incorrect. I would not feel I could be myself or be open about my relationship/sexuality in this specialty. I would not feel safe bringing my partner around other surgeons.”

A summary of themes with representative comments are reported in S2 Appendix.

## Discussion

With increasing acceptance and visibility of diverse sexual orientations and gender identities, efforts to dismantle systemic barriers to the advancement of this community have become increasingly important. Several studies from previous decades have revealed inherent bias against undergraduate medical trainees who identify as sexual and gender minorities, underscoring the need for reform [11, 12]. As the first study to date assessing medical students’ experiences and perspectives on the acceptance of SGM trainees among various medical and surgical specialties, this work made several key observations. Compared to their peers, SGM medical students more often encountered episodes of mistreatment as well as more frequently contemplated suicide. Additionally, a majority of these trainees expressed concern that their sexual orientation or gender identity would negatively impact their future career with 39.8% being previously advised to avoid disclosure. Overall, general surgery and surgical subspecialties were perceived to be the least accepting of SGM medical students, which may partially be attributable to lack of diversity in the field.

Similar to existing literature assessing medical trainees’ experiences with mistreatment, SGM respondents noted increased incidence of bullying by other students. Alarmingly, these individuals also reported more frequent thoughts of committing suicide than their peers. Utilizing the AAMC’s Graduation Questionnaire, Hill *et al*. reported that 43.5% of SGM students experienced at least one episode of mistreatment compared to 23.6% of their non-SGM counterparts [13]. In the current study, respondents identified various forms of mistreatment previously shown to exacerbate minority-stress in SGM individuals, including misgendering [23] and derogatory comments regarding sexual orientation or gender identity [24]. The burden of minority-stress has been previously noted to contribute to poor mental health and increased suicidal ideation in the general SGM population [25, 26]. These lived experiences pervade into medical training and most likely contribute to SGM trainees across the United States reporting disproportionate incidences of burnout [14] and depression [27]. To safeguard all students and foster an inclusive learning environment, individual institutions must reform to prevent mistreatment by educating both trainees and faculty as well as actively enforcing policies combatting these events.

In addition to addressing minority-stress, institutions must also acknowledge the impact of SGM identity on career trajectory. As noted, a majority of SGM trainees were found to have concerns regarding how their identities would affect future advancement with 18.5% planning to not disclose during the residency application process. Identity concealment remains a significant source of stress for SGM individuals with many opting to “pass” or “blend” into the dominant cisgender heterosexual population [23, 28]. Similar to previous work [17], SGM students surveyed feared that disclosure would consequently lead to rejection from residency programs. Though the studies that noted discrimination against SGM applicants in residency selection are decades-old [11, 12], these fears persist and remain valid. Such concerns should be addressed by both medical school and professional organization leadership as these not only negatively impact trainees’ experiences but also contribute to lack of diversity in certain fields.

Overall, respondents in the present study perceived surgery and its subspecialties as least accepting of SGM medical students. Historically, the field has been observed to hold unfavorable attitudes towards individuals of non-traditional sexual orientations or gender identities. In fact, studies from previous decades noted that 30% of surgeons expressed homophobic sentiments [29, 30] with 16% noting that they would discourage SGM trainees from entering their specialty [12]. Although surgery and its subspecialties have progressed since the publication of these studies, SGM trainees remain underrepresented and continue to face barriers not encountered by their peers [7, 8]. In 2014, Lee and colleagues found that 54% of surgical residents, regardless of their sexual or gender identity, witnessed homophobic remarks from other trainees, nurses, and attendings [17]. Of the SGM residents surveyed, a majority reported actively concealing their identity from their colleagues due to fears of rejection and discrimination.

In addition to concerns regarding discrimination on the basis of sexual orientation and gender identity, respondents in the current study cited lack of diversity as factoring into the perception that surgery is unaccepting of SGM individuals. Describing their experiences during surgical rotations, students utilized terms such as “white cis-male dominated,” “fraternity,” and “boys’ club.” Although representation of SGM identities in Medicine has yet to be studied, a large body of evidence has demonstrated the underrepresentation of women and racial minorities in academic surgical leadership positions [31–38]. Compared to other fields, surgery and its subspecialties remain the least diverse with data from the AAMC’s 2019 Diversity in Medicine Report revealing that surgeons remain predominantly white and male [39]. Lack of access to attendings and mentors of similar backgrounds may perpetuate the perception that surgery is unaccepting of SGM identity. In an effort to promote diversity, professional organizations and individual residency programs must actively recruit underrepresented individuals at all levels of training. Such efforts require internal review of each institution’s culture and commitment to diversity as well as pipeline programs and targeted recruitment for underrepresented trainees [40, 41]. Aside from addressing its history of discrimination, Medicine regardless of specialty must also transition from not only tolerating and accepting diversity but to embracing and promoting it in its various forms.

As acceptance and visibility of SGM individuals continues to grow, understanding and promoting diversity becomes of paramount importance to both medical education and patient care. Overall, undergraduate medical trainees have consistently expressed interest in receiving formal education on SGM healthcare [24, 42]. Implementation of educational interventions, such as lectures designed by physicians with expertise in the topic as well as small group sessions with SGM patient visitors, have previously been demonstrated to be effective in increasing students’ confidence in interacting and evaluating members of this community [43–45]. In addition to educating trainees about the unique challenges of this population, representation of SGM individuals across specialties must also be addressed. Assessing both trainees and practitioners, Sitkin and colleagues noted that those identifying as SGM valued role models with similar identities when choosing specialties [15]. Furthermore, increasing diversity across all specialties may improve patient experiences. Interactions with a heteronormative healthcare system as well as with providers that lack sufficient knowledge about SGM-specific issues increases stress and perpetuates healthcare disparities within this community [46]. Access to SGM or non-SGM “ally” providers may be beneficial to patient-physician interactions and thus improve healthcare in this population. Therefore, all medical and surgical specialties, particularly those in which SGM physicians are underrepresented, may benefit from efforts to increase diversity.

The present study has several important limitations inherent to the survey tool’s voluntary nature. Overall, response rate was only 12.0%, affecting our ability to generalize our findings to all actively enrolled University of California medical students. Furthermore, 26.9% of respondents identified as SGM, which may represent response bias. Naturally, SGM students are more invested in topics affecting their own community and are therefore more likely to participate. Similarly, non-SGM “ally” medical students may also have been more likely to respond to our survey. This response bias may have affected perceived SGM acceptance among specialties as well as qualitative responses provided. Finally, this survey was only made available to medical students at six University of California campuses. Further work is necessary to assess this topic across institutions. Nonetheless, this study is the first to provide insight on undergraduate medical trainees’ perspectives on SGM acceptance among select medical and surgical specialties.

Over the past several decades, Medicine has taken steps to address its history of discrimination against SGM patients and providers. However, the current study highlights the need for continued efforts to address barriers present in undergraduate medical education, specifically in general surgery and surgical subspecialties. To continue progressing, Medicine must transition from not only tolerating and accepting SGM providers and trainees but to actively promoting diversity in sexual preference and gender identity regardless of specialty.

## Supporting information

S1 Appendix Perspectives on sexual and gender minority acceptance survey. The attached survey was designed utilizing a comprehensive multistage approach with the assistance of content experts in qualitative research.
(PDF)

S2 Appendix Themes identified from qualitative responses with representative comments. Qualitative responses were assessed using a cutting-and-sorting technique by authors JM, SR, and ZT.
(DOCX)
