# Supplementary material for: Sexual and gender minority identity in undergraduate medical education: Impact on experience and career trajectory
Source: PLoS One. 2021 Nov 19;16(11):e0260387. doi: 10.1371/journal.pone.0260387 (PMC8604342; doi:10.1371/journal.pone.0260387)
Supplement: S1 Appendix — The attached survey was designed utilizing a comprehensive multistage approach with the assistance of content experts in qualitative research. (PDF) [file pone.0260387.s001.pdf]

Perspectives on LGBTQ Acceptance

Research Information Sheet

Dear UC Medical Student:

You are being asked to participate in a UCLA research study. We hope to examine how medical student perceptions of LGBTQ+ acceptance varies among the medical and surgical specialties and how LGBTQ+ identity affects career choice. Regardless of your demographics or level of training, we greatly value your input.

This is a voluntary survey that should take you less than ten (10) minutes to complete. Your responses are completely anonymous. No identifying questions will be asked and no tracking information will be collected. We are using a secure web-based application called REDCap, and only the research investigators will have access to the de-identified, collected data. Be assured that your personal data will NOT be disclosed to your respective institutions. Survey participation is completely voluntary and you may stop the survey at any point in time. We encourage you to take the survey in one sitting in a private place.

Your completion of the survey will constitute implied consent.

One (1) gift card in one full sum of $100 will be raffled to one participant if they choose to enroll in the raffle and voluntarily share their email for the sole purpose of the gift card raffle. Participation in the study is not required in order to participate in the raffle. These procedures will be followed:

1) Any individual who is asked to participate in the research study but declines, who consents/assents to enroll in the study, or who fails to complete the study, will be given equal compensation by having an equal chance of winning. Participants can email the study coordinators to be registered from the lottery.

2) Any individual who is not asked to participate in the study but wishes to be included in the lottery, raffle, and/or drawing may enter by emailing the study coordinators.

3) The winner of the gift will be selected by random number generator and will be notified via provided email. In the event that the winner does not respond after two emails, a new winner will be selected with the random number generator.

4) The approximate chance of winning is no less than 1 in 500.

If you have any questions or concerns about this study, please feel free to reach out to study investigators Sarah Rudasill (srudasill@mednet.ucla.edu) or Josef Madrigal (josefmadrigal@mednet.ucla.edu). This research study is conducted under the Principal Investigator Dr. Peyman Benharash, Associate Professor-in-Residence of Surgery and Bioengineering, of the Department of Cardiac Surgery at UCLA. If you have questions about your rights as a research subject, or you have concerns or suggestions and you want to talk to someone other than the researchers, you may contact the UCLA OHRPP by phone: (310) 206-2040; by email: participants@research.ucla.edu or by mail: Box 951406, Los Angeles, CA 90095-1406.

Demographic Data

Which of the following is your University of California (UC) campus?	◯ Davis<br>◯ Irvine<br>◯ Los Angeles<br>◯ Riverside<br>◯ San Diego<br>◯ San Francisco
Which of the following best describes your year in medical school?	◯ MS1<br>◯ MS2<br>◯ MS3<br>◯ MS4<br>◯ Leave of Absence (LOA)<br>◯ Other

What is your age in years?	__________________________
Which most closely describes your race? (Please check all that apply)	☐ American Indian or Alaska Native<br>☐ Asian (including Indian subcontinent and Philippines origin)<br>☐ Black or African American<br>☐ White (including Middle Eastern origin)<br>☐ Native Hawaiian or Other Pacific Islander<br>☐ Other
If you selected other for race, feel free to write-in your race here.	________________________________
Which the following best describes your relationship status?	◯ Single<br>◯ In a relationship<br>◯ Other
Which of the following is the best estimate of your parents' household income in the last year?	◯ $0 - $50,000<br>◯ $50,000 - $100,000<br>◯ $100,000 - $250,000<br>◯ >$250,000
What is your gender identity**?<br>**Gender Identity: A person's deeply felt psychological identification as male, female, transgender, no gender, or another gender, which may or may not correspond to the person's body or designated sex at birth.	◯ Female<br>◯ Male<br>◯ Transgender<br>◯ Nonbinary<br>◯ Other<br>◯ Decline to answer
If you answered other, please feel free to elaborate in the following space:	________________________________
What is your sexual orientation**?<br>**Sexual orientation: An individual's self-identified state of physical and/or emotional attraction.	◯ Bisexual<br>◯ Gay / Lesbian<br>◯ Heterosexual<br>◯ Queer<br>◯ Other<br>◯ Decline to answer
If you answered other, please feel free to elaborate in the following space:	________________________________
What is your intended specialty or current specialty of interest?	◯ Anesthesiology<br>◯ Emergency Medicine<br>◯ Family Medicine<br>◯ Internal Medicine<br>◯ Neurology<br>◯ Obstetrics and Gynecology<br>◯ Pediatrics<br>◯ Psychiatry<br>◯ Surgery / Surgical Subspecialties<br>◯ Undecided<br>◯ I will not be applying into residency<br>◯ Other: (write in)

If you answered other, please write-in your specialty of choice: ________________________________

Specialty-Specific Questions

Based on your own perceptions, how accepting of LGBTQ students are attending physicians in the following specialties?

0 - Never Accepting, 50 - Sometimes Accepting, 100 - Always Accepting

Anesthesiology

Never Accepting — Sometimes Accepting — Always Accepting

(Place a mark on the scale above)

Emergency Medicine

Never Accepting — Sometimes Accepting — Always Accepting

(Place a mark on the scale above)

Family Medicine

Never Accepting — Sometimes Accepting — Always Accepting

(Place a mark on the scale above)

Internal Medicine

Never Accepting — Sometimes Accepting — Always Accepting

(Place a mark on the scale above)

Neurology

Never Accepting — Sometimes Accepting — Always Accepting

(Place a mark on the scale above)

Obstetrics and Gynecology

Never Accepting — Sometimes Accepting — Always Accepting

(Place a mark on the scale above)

Pediatrics

Never Accepting — Sometimes Accepting — Always Accepting

(Place a mark on the scale above)

Surgery

Never Accepting — Sometimes Accepting — Always Accepting

(Place a mark on the scale above)

If you responded "never accepting" to one or more of the above fields and wish to elaborate on your reasons or experiences, please do so here: ____________________________________

Specialty-Specific Questions

How comfortable would you be applying for residency in the following specialties?

0 - Never Comfortable, 50 - Sometimes Comfortable, 100 - Always Comfortable

Anesthesiology

Never Comfortable | Sometimes Comfortable | Always Comfortable

(Place a mark on the scale above)

Emergency Medicine

Never Comfortable | Sometimes Comfortable | Always Comfortable

(Place a mark on the scale above)

Family Medicine

Never Comfortable | Sometimes Comfortable | Always Comfortable

(Place a mark on the scale above)

Internal Medicine

Never Comfortable | Sometimes Comfortable | Always Comfortable

(Place a mark on the scale above)

Neurology

Never Comfortable | Sometimes Comfortable | Always Comfortable

(Place a mark on the scale above)

Obstetrics and Gynecology

Never Comfortable | Sometimes Comfortable | Always Comfortable

(Place a mark on the scale above)

Pediatrics

Never Comfortable | Sometimes Comfortable | Always Comfortable

(Place a mark on the scale above)

Surgery

Never Comfortable | Sometimes Comfortable | Always Comfortable

(Place a mark on the scale above)

If you responded "never comfortable" to one or more of the above fields and wish to elaborate on your reasons or experiences, please do so here: ______________________

Mental Health / Work Environment Questions

When answering these questions, please reflect on the time from when you started medical school to the present. Considering your interactions as a medical student, how frequently have you experienced the following?

(0 Never, 50 Sometimes, 100 Always)

Witnessed homophobic/transphobic remarks either to you or to someone else?

Never Sometimes Always

(Place a mark on the scale above)

If you have witnessed homophobic/transphobic remarks, who has been the source? Check all that apply

☐ Medical Students ☐ Residents ☐ Attending Physician Tutors (PBL, Clinical Skills, Doctoring) ☐ Attending Physician Clinical Supervisors ☐ Nurses ☐ Other Hospital Staff

Witnessed discrimination?

Never Sometimes Always

(Place a mark on the scale above)

Felt bullied by fellow medical students?

Never Sometimes Always

(Place a mark on the scale above)

Felt bullied by a resident or an attending?

Never Sometimes Always

(Place a mark on the scale above)

Felt bullied by other hospital staff?

Never Sometimes Always

(Place a mark on the scale above)

Contemplated leaving medical school?

Never Sometimes Always

(Place a mark on the scale above)

Contemplated suicide?

Never Sometimes Always

(Place a mark on the scale above)

If you endorsed experiencing any of the scenarios above and wish to share a de-identified example, please do so here:

____

Have you ever been advised to avoid disclosing your gender identity or sexual orientation?

◯ Yes ◯ No ◯ Decline to answer

If you have been advised against disclosure, please feel free to elaborate on the circumstances:

____

Do you have any concerns that disclosure of your gender identity or sexual orientation would risk your future career?	◯ Yes ◯ No ◯ Decline to answer
If you have concerns about how disclosure may affect your future, please feel free to elaborate on your concerns:	__________________________________
Have you ever taken steps to hide your orientation or identity during medical school?	◯ Yes ◯ No ◯ Decline to answer
If so, please feel free to elaborate on your efforts:	__________________________________
Do you plan to disclose your gender identity or sexual orientation when you apply for residency?	◯ Yes ◯ No ◯ Undecided
If you do not plan to disclose your gender identity or sexual orientation during the residency application process, which of the following reasons (if any) factor into your decision?	☐ Lack of a supportive environment in medical school ☐ Fear of discrimination in the application process<br>☐ Fear of discrimination in residency<br>☐ Concern over future career options<br>☐ Advice from mentors or faculty<br>☐ Pressure from family or friends<br>☐ Cultural or social norms<br>☐ Religious beliefs ☐ Nobody's business<br>☐ Decline to answer ☐ Other reason
If you wish, please share more information about your barriers to disclosure in the residency application process:	__________________________________
If you would like to be entered into the raffle for a $100 gift card, please provide your university-issued email address here. Note that your responses are not identifiable and will be kept separate from your email.	__________________________________
