# Supplementary material for: Sexual and gender minority identity in undergraduate medical education: Impact on experience and career trajectory
Source: PLoS One. 2021 Nov 19;16(11):e0260387. doi: 10.1371/journal.pone.0260387 (PMC8604342; doi:10.1371/journal.pone.0260387)
Supplement: S2 Appendix — Qualitative responses were assessed using a cutting-and-sorting technique by authors JM, SR, and ZT. (DOCX) [file pone.0260387.s002.docx]

**S2 Appendix. Themes Identified from Qualitative Responses with Representative Comments**

| **Theme 1:** Specialties Viewed as Less Accepting Lack Diversity |
| --- |
| - Personally, I feel like fields that are mostly white cis-male dominated and “competitive” specialties seem to be way less accepting of those that are different than the status quo. |
| - I think that surgery is considered never accepting because the field has a very patriarchal and hierarchal system. In my experience, there is less acceptance towards women, non-white and LGBTQ folk – anyone who is outside the stereotype. |
| - Many surgical specialties are male-dominated and tend to primarily be white males. The most homophobic and transphobic comments I have ever witnessed were during surgical rotations. |
| - Rotating through surgery felt like being part of a fraternity, and it was hard to connect with the “bros.” They never showed interest in engaging with me because we had little in common. They did their own thing and left me on my own. |
| - Surgery feels like a “boys club.” In California, it’s probably more accepting of less heteronormative individuals and LGBTQ folks, but the sentiment still exists. I think it’s also sub-specialty dependent (i.e. Ortho-Bros). |
| - Cis-male dominated fields just seem more uncomfortable to pursue as a BIPOC woman. |
| - I do not see my own identity or beliefs reflected in attendings and residents in surgery. Surgical culture tends to be blunt and often politically incorrect. I would not feel I could be myself or be open about my relationship/sexuality in this specialty. I would not feel safe bringing my partner around other surgeons. |
| - Surgery is too hierarchical for my comfort level. It is also straight male dominated, so I don’t think I would fit in. |
| - A gay black woman isn't necessarily granted much space in the white cis male world of medicine. As someone who hopes to land in positions of leadership, sharing information about my partner when it arises in conversation has always given me pause. But I'm hopeful the emotional tides will shift, not only with society, but my own personal levels of comfort. |

| **Theme 2:** Students Witnessed or Experienced Misgendering |
| --- |
| - I think it’s hard to determine whether these fields are accepting of “LGBTQ” people broadly when there is a huge difference. Most specialties have been accepting of my “sexuality” but never get my pronouns right or make any effort to respect my gender identity. |
| - As a non-binary queer person, some of my most uncomfortable moments in medical school were during my Ob/Gyn rotation. I heard providers repeatedly mis-gender non-binary and trans patients, who were pregnant, and also make disparaging comments about individuals seeking gender-affirming surgical procedures. There were definitely some physicians who were wonderful queer/trans allies, but the overall environment was very unwelcoming to LGBTQIA+ individuals. |
| - Don’t know if this counts, but I get mis-gendered all the time even when my pronouns are clearly visible. I often feel out of place, which has contributed to my thoughts about leaving medical school. My suicidal ideation is not related to my gender identity or sexual orientation. |
| - Residents outright refusing to use appropriate pronouns for trans patient (pediatrics) or repeatedly and negligently forgetting to use the correct pronouns (most specialties). |
| - An attending physician at an outpatient clinic constantly mis-gendered an adolescent patient despite me telling him the patient’s preferred pronouns at least twice in my presentation. |
| - When I was shadowing a plastic surgery, the surgeon and residents in the OR were playing a racist song on YouTube (a spoof of a popular song with the lyrics replaced) about how Asians eat a lot of rice. To me, they said, "You're not going to tell anyone about this right?" because I was a student. And then they went on to discuss how pronouns are a part of everyone's badges now and how it's too much work to remember people's pronouns. I REALLY disliked that experience and I would never want to experience something like that again. |
| **Theme 3:** Attendings, Residents and Other Hospital Staff Made Explicit Comments Regarding Individuals of SGM Identity |
| - I witnessed some surgeons be very supportive, including having the opportunity to be part of a gender affirming surgery on urology. However, others on trauma and general surgery would make derogatory jokes (anti-LGBT and racist), making fun of pronouns, sexual behaviors, and partners who were present. I honestly think this would correlate with burn out. The more sleep deprived the service, the worse the behavior. |
| - Because people typically assume I’m heterosexual, sometimes they feel comfortable making jokes or comments that are homophobic in my presence. Not about me, but about others. I am currently in a heterosexual relationship and typically do not disclose my orientation (bisexual) unless I really trust the person and the topic specifically comes up. Out of my good friends in medical school, I’ve probably told less than 5%. |
| - Attending Physician in Ob/Gyn: LAUGHED OUT LOUD after making a homophobic joke. |
| - I had an LGBT patient there for severe fungal infection of the face, and jokes were made about their sexual behavior, implying it resulted in the infection. |
| - Nurses in the OR made fun of my pride flag pin on my badge. |

| **Theme 4:** SGM Medical Students View Identity as Barrier to Future Career |
| --- |
| - At multiple residency application advising sessions, there was mixed opinion on whether LGBT students should reveal their sexual orientation, either through personal statements or by disclosing certain extracurricular activities. Faculty noted that certain conservative specialties (including surgery) in conservative locations (Southern States) may use this as a mark against you. |
| - I was told that, since I am hetero passing due to the nature of my long-term relationship, it is best for me to not disclose as it may affect other's view of me and my opportunities |
| - In the residency application process, I was advised by a mentor from a different institution to not mention my same-sex partner on the interview trail. I considered this but ultimately decided against concealing this part of my identity during the process. |
| - When applying to medical school, I was advised not to disclose gender/ sexual orientation in my personal statement because I was told that it might work against me when applying to more "conservative" programs |
| - I'm bisexual, but typically date cis, heterosexual males and so it is very easy for me to be perceived as a cis, heterosexual female, thus I typically don't disclose my sexual orientation during applications/interviews both because I don't feel a strong need and because I know it would just introduce opportunity for bias and discrimination. |
| - We live in a patriarchal, heteronormative white supremacist society. And because of that, I've been advised that because I pass as heterosexual, people in positions of power (who are often straight cis-gender white men) may see me as being in their “in-group” and feel more comfortable supporting me than if I disclosed that I wasn't as like them as they think. |
